# Managing distress on an inpatient adolescent ward

**DOI:** 10.1186/2050-2974-3-S1-O46

**Published:** 2015-11-23

**Authors:** Colleen Alford, Annaleise Robertson, Jane Miskovic-Wheatley, Andrew Wallis

**Affiliations:** 1 The Children's Hospital at Westmead, Eating Disorder Service, Sydney, Australia

## 

The Children's Hospital at Westmead Eating Disorder Service provides secondary and tertiary level care to inpatients and outpatients. The inpatient program focuses on medical stabilisation, re-establishing eating, and containing eating disorder behaviours as well as assessing and managing comorbid problems such as anxiety, depression or risk behaviours. Being in hospital and being faced with a healthy amount of food and restrictions around exercise can be distressing for these inpatients. The team at CHW has developed a Distress Management Plan to ensure that patients receive timely and appropriate support. This includes education, coordination of team input and resources, clear and consistent documentation, the provision of therapeutic support and strategies. This oral presentation will discuss the Distress Management Plan and its implementation as well as the therapeutic strategies covered. Data on its effectiveness around patient and staff experience and overall outcome will also be discussed.

